# Human Intestinal Microbiota-Derived Short-Chain Fatty Acid and Lactate Profiles In Vivo and In Vitro: A Structured Review and Comparative Case Study

**DOI:** 10.3390/nu18213701

**Published:** 2026-11-07

**Authors:** Agnieszka Rudzka, Ondřej Patloka, Marcin Kruk, Magdalena Płecha, Marek Zborowski, Tomasz Królikowski, Michał Oczkowski, Danuta Kołożyn-Krajewska, Marcelina Karbowiak, Wioletta Mosiej, Renata Barczyńska-Felusiak, Dorota Zielińska

**Affiliations:** 1 Department of Dietetics and Food Studies, Faculty of Science and Technology, Jan Dlugosz University in Czestochowa, Al. Armii Krajowej 13/15, 42-200 Częstochowa, Poland; d.kolozyn-krajewska@ujd.edu.pl (D.K.-K.); r.barczynska-felusiak@ujd.edu.pl (R.B.-F.); 2 Department of Food Technology, Faculty of AgriSciences, Mendel University in Brno, Zemědělská 1, 61300 Brno, Czech Republic; xpatlok3@mendelu.cz; 3 Department of Gastronomy and Food Health Quality, Faculty of Health Sciences, Warsaw University of Life Sciences (WULS-SGGW), Nowoursynowska 159 C, 02-776 Warsaw, Poland; marcin_kruk@sggw.edu.pl (M.K.); marcelina_karbowiak@sggw.edu.pl (M.K.); wioletta_mosiej@sggw.edu.pl (W.M.); 4 Institute of Biochemistry and Biophysics, Polish Academy of Sciences, Pawińskiego 5a, 02-106 Warsaw, Poland; mk.plecha.ibb@gmail.com; 5 Institute of Evolutionary Biology, Faculty of Biology, Biological and Chemical Research Centre, University of Warsaw, Żwirki i Wigury 101, 02-089 Warsaw, Poland; 6 The Faculty of Medicine and Health Sciences, University of Applied Sciences in Nowy Sącz, Kościuszki 2G, 33-300 Nowy Sącz, Poland; mzborowski@ans-ns.edu.pl; 7 Department of Dietetics, Faculty of Health Sciences, Warsaw University of Life Sciences (WULS-SGGW), Nowoursynowska 159 C, 02-776 Warsaw, Poland; tomasz_krolikowski@sggw.edu.pl (T.K.); michal_oczkowski@sggw.edu.pl (M.O.)

**Keywords:** Microbiota, dynamic in vitro simulation, short-chain fatty acids, probiotic intervention, dietary intervention

## Abstract

**Background**: The transfer of human intestinal microbiota from in vivo to in vitro conditions alters its structure. This transition may trigger functional changes in the microbial community, such as its ability to produce short-chain fatty acids (SCFAs) and lactate. **Methods**: This study evaluated SCFAs and lactate profiles in the dynamic simulator and in the feces of a human volunteer who donated a sample for the inoculation of the system. Changes in SCFA and lactate levels were monitored over a 28-day dietary intervention and a parallel 14-day probiotic supplementation. **Results:** Microbial and dietary variation were not significant predictors of variability in SCFA and lactate profiles. In contrast, culturing environment and probiotic intervention exerted significant effects on SCFAs and lactate. The probiotic intervention appeared to influence SCFA and lactate profiles under in vitro conditions, whereas no similar effect was observed in vivo. Furthermore, the abundance of individual microbial genera was mostly weakly correlated with SCFA and lactate levels, and the direction of these correlations was inconsistent across two microbiota culturing environments. **Conclusions:** Our findings suggest that structural changes in the microbiota during its transfer from in vivo to in vitro conditions are accompanied by alterations in microbial functions, such as SCFA and lactate levels or response to interventions. However, these responses align with data from other human studies, supporting the ability of dynamic in vitro models to simulate key functions of the intestinal microbiota. Further optimization is required to accurately model personalized microbiota responses.

## 1. Introduction and a Structured Review

### 1.1. Rationale Behind the Review and Its Aim

Organic acids are important metabolites of human intestinal microbiota [1,2]. In recent decades, researchers have broadly studied their concentrations and profiles in human-derived samples, leading to interest in establishing reference values [3]. While human and animal studies provided the basis for linking the organic acid profiles to health outcomes, in vitro reports were indispensable in revealing underlying mechanisms [1]. Therefore, it is important to establish how reliably organic acid profiles may be simulated during human microbiota culture in vitro.

This structured review aimed to evaluate the extent to which the organic acid levels measured in dynamic in vitro microbiota cultivation simulators (DIVMSs) reflect in vivo conditions, and how such measurements should be interpreted. The review begins with a short introduction to the medical implications of organic acid levels in the human body, then describes mechanisms by which these levels might be influenced based on the findings from both in vitro and in vivo studies. Finally, it provides a broader overview of the capabilities of current dynamic human microbiota simulators and the translational gaps that require addressing.

### 1.2. Organic Acids as Human Microbiota Metabolites

Short-chain fatty acids (SCFAs), primarily acetate, propionate, and butyrate, together with lactate, are among the most important metabolites produced during the fermentation of nondigestible carbohydrate fractions by the gut microbiota and are recognized as indicators of the metabolic activity of intestinal microorganisms [4]. SCFAs play a key role in maintaining gastrointestinal homeostasis by serving as the primary energy source for colonocytes, aiding their proliferation, strengthening intestinal barrier integrity, and participating in the regulation of immune responses and host metabolic processes [2,5]. Lactate, in turn, acts as an important intermediate metabolite that is utilized by bacteria involved in cross-feeding interactions and can serve as a substrate for the production of butyrate and other SCFAs, thereby contributing to the stability of the gut microbial ecosystem [4]. SCFAs and lactate are frequently quantified in fecal samples, where they represent the end products of microbial fermentation and reflect the metabolic activity of the gut microbiota [4,6]. The SCFAs can positively regulate human metabolism, may participate in appetite regulation and even suppress hepatic gluconeogenesis and lipogenesis [7]. In recent years, reference ranges for SCFA concentrations in feces and plasma of healthy individuals have been established, which may facilitate the interpretation of research findings and support their application as biomarkers of health and disease [3]. The interpretation of SCFA concentrations, however, requires caution, as it is influenced by multiple factors, including dietary habits, gut microbiota composition and its functional and metabolic activity, intestinal absorption or intestinal transit time [8,9]. Moreover, elevated fecal SCFA concentrations do not necessarily reflect beneficial microbial metabolic activity and have been associated with obesity, hypertension, and other cardiometabolic risk factors [10].

### 1.3. Drivers of Organic Acid Production by the Microbiota and In Vitro Simulation

In vitro models are used throughout the literature to study microbiota responses to various interventions. The ease of sampling and standardized microbiota culturing conditions allow for the elimination of some of the confounding variables that impede data interpretation in human studies, making DIVMSs particularly useful for addressing mechanistic questions. One of the most widely researched subjects, besides changes in microbiota composition, is also the production of SCFAs in response to various interventions. Endogenous SCFA production in humans can be enhanced through dietary interventions that modulate the composition and activity of the gut microbiota, including adequate intake of dietary fiber, prebiotics, as well as selected probiotic interventions, which may stimulate the microbial synthesis of these metabolites [8,11,12]. Fermentable substrates determine the maximum potential yield of SCFAs. The main substrates for SCFA production include dietary fiber and resistant starches, collectively referred to as microbiota-accessible carbohydrates [13]. Microbiota-accessible carbohydrates that reach the colon are initially converted mainly into lactate and acetate by primary fermenters. Subsequent cross-feeding interactions promote the formation of propionate and butyrate. Consequently, the chemical nature of the substrate largely determines the resulting SCFA profile [14,15]. Comparable diet-driven modulation has been demonstrated repeatedly in the simulator for prebiotic, human-milk-oligosaccharide and protein substrates, where supplementation reproducibly raised total SCFAs and shifted molar ratios in favor of propionate or butyrate [16,17,18]. Probiotic strains act through a narrower and more context-dependent mechanism. Quantitative analyses of commonly used probiotic strains indicate that, in monoculture, they produce mainly acetate and lactate. Only a limited number of species are capable of generating appreciable amounts of propionate or butyrate. Consequently, the direct contribution of probiotics to the overall SCFA pool is generally modest [19]. Their more important role is indirect: they supply lactate to propionate- and butyrate-forming partners such as *Veillonella* and members of the *Lachnospiraceae*, thereby reshaping community output [18,20].

The most popular and standard DIVMS approaches do not simulate host absorption of microbial metabolites [21], allowing them to accumulate in the reactor medium. Consequently, such an approach may help to understand whether a given intervention increases overall SCFA concentration available for uptake in the colon. This is an important difference between in vivo and in vitro studies, since in humans approximately 95% of total SCFAs are absorbed in the human intestine and only about 5% is excreted in the feces [22]. Several authors have used DIVMSs to monitor changes in SCFA concentrations. For example, Wu et al. (2019) showed that replacement of the standard feed with media based on cooked grains (rice as the control diet and the intervention being a traditional Chinese medicine dietary therapy based on wholegrains with the addition of lily and *Ganoderma lucidum* polysaccharides) resulted in a many-fold increase in acetate, butyrate and propionate levels in the liquid drawn from DIVMS bioreactors [23]. Others applied specific substances into standard media. Substances such as human milk oligosaccharides (fucosyllactose and sialyllactose), pomegranate extracts, or oat products were added, giving appreciable increases in the SCFA concentrations in adult human microbiota cultures [18,24,25,26].

### 1.4. Validation, Stabilization and Metabolic Concordance Between In Vitro and In Vivo Microbiota Studies

The first validation studies of the DIVMS used SCFA profiles as the main outcome for determining the microbiota stability and also compared their relative proportions with SCFA profiles reported in human studies, showing a good coverage [27,28]. Further dietary and antibiotic-therapy interventional studies showed that SCFA profiles in the DIVMS distal colon compartment aligned well with data collected in studies measuring SCFA content in human feces [29]. Therefore, the approach allows for an accurate reflection of the SCFA profile compared to in vivo data, providing a means for studying the metabolic functions of microbial communities under controlled conditions. However, it is also known that each individual microbiota has a different capacity for SCFA production. This is a well-documented fact from human and also in vitro studies utilizing DIVMSs [4,6,25,30,31]. The transfer of microbiota from the host to the in vitro environment inevitably leads to structural and functional changes in microbial community, particularly during the initial stabilization phase of the reactor [32]. Therefore, assessing the extent to which SCFA and lactate profiles obtained in such systems reflect individual in vivo conditions is crucial. Reactor stabilization is a recognized prerequisite before any interventional readout, yet the transition from the gut lumen to a continuous bioreactor consistently reshapes both community structure and fermentative output. During the initial adaptation of DIVMS derivatives, oxygen gradients, fixed hydraulic retention times, pH clamping and a chemically defined nutritional medium progressively select for fast-growing, medium-adapted taxa, so that the stabilized consortium diverges compositionally from the donor inoculum while retaining its principal catabolic guilds [29,33,34]. Typically, obligate anaerobes and fastidious mucosa-associated species decline, whereas facultative and lactate-handling genera such as *Streptococcus*, *Veillonella* and former genus *Lactbacillus* are relatively enriched during the early adaptation window [35]. A characteristic early signature is therefore a transient surge in lactate accumulation driven by primary fermenters, followed by cross-feeding towards acetate, propionate and, more slowly, butyrate as secondary fermenters re-establish [20,35]. This lactate-to-SCFA hand-off, and the frequently delayed recovery of butyrogenesis, largely explain the metabolic discrepancies reported between reactor effluent and paired fecal samples: absolute SCFA concentrations in vitro often exceed physiological fecal values because there is no host absorption or epithelial uptake, whereas molar ratios tend to converge on the acetate-dominant profile observed in vivo once stabilization is reached [15]. In healthy adults, acetate typically accounts for approximately 50–70% of total fecal SCFAs, with propionate and butyrate contributing roughly 15–25% each, providing a useful benchmark for evaluating the physiological relevance of in vitro fermentation profiles [36]. Because lactate is a transient intermediate rather than a terminal product, its standing pool is especially sensitive to the balance between producing and consuming populations, making it a sensitive early indicator of incomplete stabilization [20]. This suggests that a stabilization period of one to two weeks, confirmed by stable SCFA production and community composition rather than by elapsed time alone, should precede any interventional phase, and that baseline drift should be explicitly accounted for when interpreting treatment effects [29].

### 1.5. Translational Challenges in Microbiota Modeling

Although numerous studies have described associations between dysbiosis and disease, they have provided limited causal and individually predictive insights. Similarly, interventions targeting the microbiome, including prebiotics, probiotics and fecal microbiota transplantation, often produce variable or modest clinical outcomes [37]. Dynamic in vitro models occupy a useful intermediate position between reductionist laboratory approaches and complex human studies. By isolating the microbial community from host physiology, these systems enable controlled and repeated mechanistic experiments that are difficult or impossible to perform in humans [38]. Importantly, the observation that in vitro responses often follow the same general direction as those reported in human studies supports their use as functional surrogates rather than merely compositional representations of the microbiota [29,39,40,41]. However, this isolation also limits the predictive capacity of in vitro models. Important host-related factors, including metabolite absorption, immune and epithelial interactions, intestinal motility, transit time and the presence of specific mucosal niches, are absent or only partially reproduced [42]. These factors contribute to the attenuation of interventional effects observed in vivo and may explain why responses measured in vitro do not always directly translate to the host [33]. Closing the translational gap will require moving beyond single-factor experimental designs.

### 1.6. The Hypothesis, Design and the Aim of the Case Study

The compositional shifts in microbiota following inoculation and stabilization of the DIVMS may also result in changes to organic acid profiles. To test this hypothesis, we have designed a study in which SCFA and lactate levels were measured in parallel in the distal colonic DIVMS liquid and in the feces of a volunteer who was at the same time a microbiota donor for the DIVMS. Both the DIVMS and the volunteer were given a parallel dietary and probiotic intervention, allowing comparison not only of organic acid concentrations and their relative profiles, but also of changes occurring in response to the interventions.

Overall, the aim of this work was to present a unique case study where the concentration of SCFAs from a single donor’s microbiota was monitored in parallel in in vivo and in vitro environments and compared during probiotic and dietary intervention.

## 2. Materials and Methods

### 2.1. Literature Search and Study Selection

A structured literature search was conducted on 11 July 2026 in PubMed/MEDLINE, Scopus, and Web of Science Core Collection using combinations of the following keywords: gut microbiota, intestinal microbiota, in vitro, dynamic in vitro, SHIME, continuous culture, short-chain fatty acids, SCFA, acetate, propionate, butyrate, lactate, diet, dietary intervention, prebiotic, and probiotic. The search strategy was adapted to each database.

Records were independently screened by two researchers in the Rayyan. application (Rayyan Systems, Inc., Cambridge, MA, USA). Titles and abstracts were screened first, followed by full-text assessment of potentially relevant studies. Disagreements were resolved through discussion.

Studies were included if they investigated human intestinal or fecal microbiota in dynamic or controlled in vitro models and/or assessed SCFA or lactate production, dietary or probiotic interventions. Studies focusing exclusively on non-human microbiota, isolated bacterial strains without a microbial community context, or unrelated outcomes were excluded.

### 2.2. Case Study Design

A comprehensive description of the study design and methodological details are available from previously published articles [17,34,40] describing the experiment that led to the currently presented results, including a supplementary subset of the data [43,44,45,46].

Briefly, the instrumental in vitro model Simulator of the Human Intestinal Microbial Ecosystem (SHIME, ProDigest, Gent, Belgium) was run in MultiSHIME mode under standard conditions as recommended by the SHIME’s manufacturer. This study focused on the distal colon compartments with intestinal fluid in three L-SHIME bioreactors. The system is referred to as the DIVMS throughout Section 2, Section 3, Section 4 and Section 5.

The DIVMS was inoculated with a stool sample from a 39-year-old healthy female single donor. Before the experiment, the DIVMS was stabilized for 14 days using the standard protocol and recommended standard DIVMS feed medium. After stabilization, a 28-day experimental intervention was performed in the DIVMS (in vitro) as well as in human volunteer (in vivo) conditions.

Applied intervention included a parallel combined 14-day probiotic supplementation of *Lacticaseibacillus rhamnosus* GG (LGG, collection number: ATCC 53103) and a nutritional intervention for both the DIVMS and human volunteer throughout the entire 28-day experimental period. The LGG was a commercially available supplement with a content of 6 × 10^9^ CFU per capsule administered twice daily.

The dietary intervention was designed to reflect a similar nutritional composition of the volunteer’s experimental diet and the DIVMS’s feed media. Therefore, the proportion of nutrients in the standard DIVMS medium was adjusted to mimic the dietary residues that may reach the colon of the volunteer. The content of indigestible residues was calculated based on a study where the percentage residues of dietary nutrients in human ileostomies were reported [47]. Detailed information about experimental diet composition and concentration of the modified DIVMS’s feed media components is available and summarized in open data resources [44].

**Figure 1:**
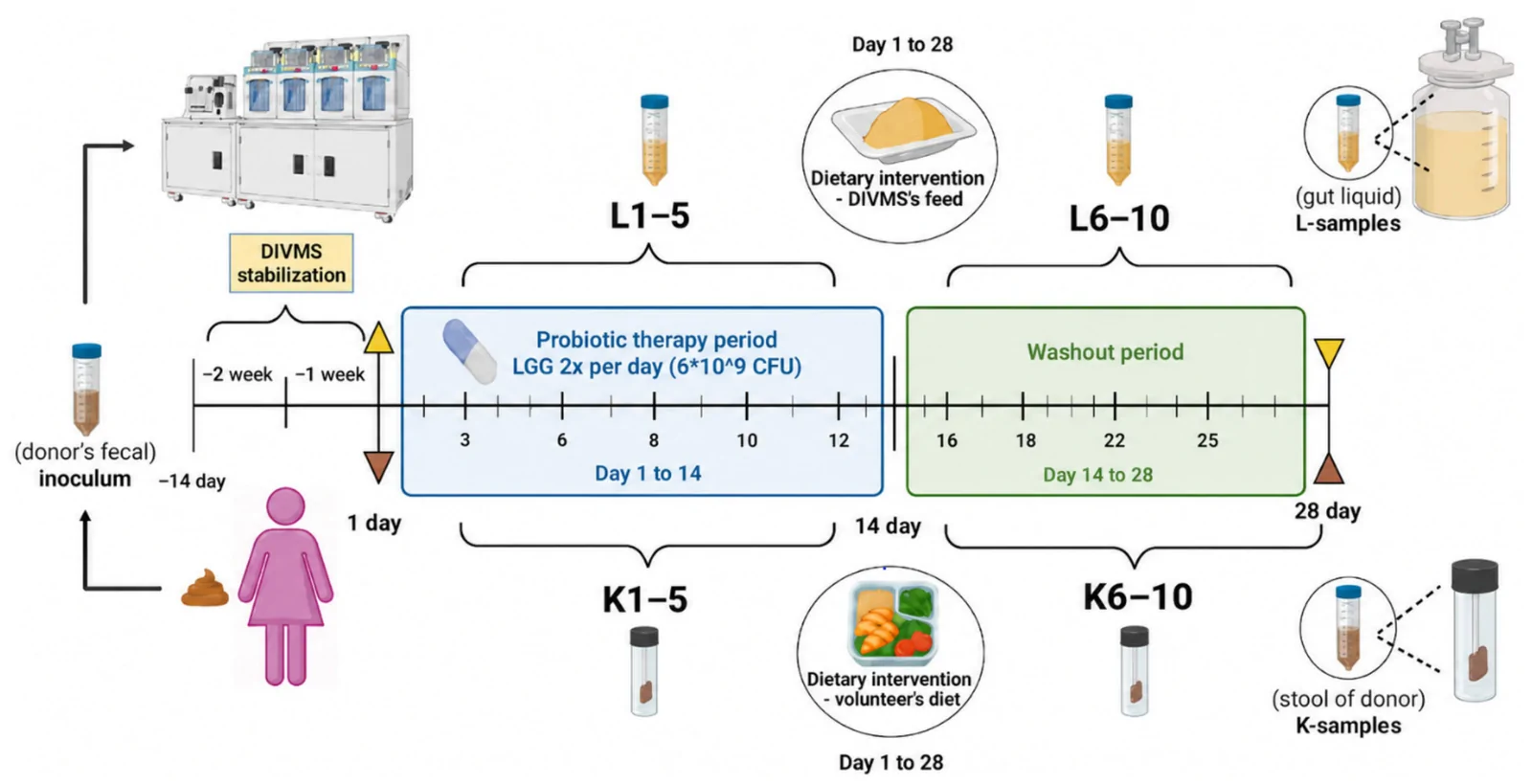
A schematic representation of the study design and sampling scheme. The figure was created with Biorender.com.

Overall, ten samples were taken during the whole experiment from both the DIVMS (liquid samples from bioreactors denoted as L1–10) and from the human volunteer (stool samples denoted as K1–10). Five samples were collected during combined probiotic and dietary intervention on experimental days 3, 6, 8, 10, and 12, marked as samples L1–5 and K1–5, respectively. The remaining samples were collected during the dietary intervention alone on experimental days 16, 18, 22, 25, and 28, marked as samples L5–10 and K5–10, respectively. The study design and sampling scheme are summarized in Figure 1. This study was approved by the Research Ethics Committee at Jan Dlugosz University in Czestochowa before the experimental work was commenced. Written informed consent was signed by the volunteer participating in the study. The blank consent form in the original Polish version and its English translation are provided in an open repository [46].

This study comprised a single experimental run with repeated sampling. Samples collected at successive time points represent repeated longitudinal observations rather than independent biological replicates.

The current study builds on the microbial abundance and nutrient intake data presented in our previous publications [17,34,40] by correlating them with SCFA and lactate content measured and reported for the first time in the current study. This approach provides new insights into microbiota functions following transfer from in vivo to in vitro conditions.

### 2.3. Preparation and Analysis of Samples

Kruk et al.’s (2025) approach was used to analyze butyrate, acetate and propionate (SCFA) as well as lactate content [48]. Samples were diluted in water (200 mg/5 mL) and sonicated for 30 min at 30 °C and 35 kHz (IS-6; Inter Sonic; Olsztyn, Poland), then centrifuged (Eppendorf Centrifuge 5804 R; Hamburg, Germany, 10,000 rpm, 10 °C, 10 min). The sonication and centrifugation were repeated once more. The supernatant was then filtered through a 0.22 μm PES syringe filter into the vials. SCFAs and lactate were analyzed using Shimadzu HPLC with LC-20 CE pump, CBM-20 A controller, CTD-20 AC oven, SIL-20 AC autosampler and UV/Vis SPD-20AV detector (Shimadzu, Kyoto, Japan). An Aminex HPX-87H column 300 × 7.8 mm (Bio-Rad, Warsaw, Poland) was used with an isocratic flow of 0.5 mL/min of 10 mM H_2_SO_4_ at 40 °C. Quantification was based on detection at 210 nm wavelength UV/Vis for organic acids and external standard curves of 0.05–80 μg per injection of each analyte. All external standards were purchased from Sigma Aldrich (Poznan, Poland) with a purity of 99%. This methodology was based on protocols used in multiple previous studies where SCFAs were analyzed in human and animal fecal samples as well as in in vitro fecal fermentation systems [48,49,50,51,52,53,54]. It has been internally validated in our laboratory across a broad range of matrices, including samples from various in vitro gastrointestinal fermentation systems and fecal samples from rats and pigs, allowing an accurate analysis of SCFA and branched-chain fatty acid profiles. The internally validated method demonstrated excellent linearity (R^2^ ≥ 0.99) over an analytical range extending from the limit of quantification (LOQ; 50–100 ng) to 55 µg per injection, with recoveries ranging from 92% to 105% and relative standard deviations (RSDs) of 3–19%.

The DNA was extracted from all collected samples, and the compositional changes in the microbiota were assessed by means of high-throughput sequencing of the V3–V4 regions of 16S rRNA according to protocols described in our previous publication [34]. Results of microbiota compositional analysis were reported previously [40,44], and these were used for statistical output reported in Section 3.2 of the current manuscript.

### 2.4. Statistical Evaluation

Statistical analyses were performed in R software version 2026.05.0 +218 (Posit, Boston, MA, USA). The compositional data of microbial abundance were centered log-ratio (CLR)-transformed, and for SCFA, lactate and nutrient concentrations, z-score standardization was applied (separately for volunteer and DIVMS data) before analyses. Three outlying samples for each dataset (DIVMS: L3, L4 and L10 and the volunteer: K3, K4 and K10) that were identified in the study on the microbiota composition [40] were removed from the statistical comparisons. Transformed, normalized, and outlier-free data were then subjected to statistical analyses.

In preparation for the main analysis, principal component analyses (PCAs) were performed. There were three PCAs performed: the first for SCFA + lactate concentrations, the second for nutrients in DIVMS media and calculated residues that could have reached the volunteer’s colon (animal and non-animal protein, sugars and soluble fiber), and the third for microbiota at the phylum level giving SCFA + lactate, nutrient and micro principal components (PCs) respectively. Obtained PCs were used in the two following linear models, constituting the main analysis:

Model 1 (microbiota model):

SCFA + lactate_PC1 and SCFA + lactate_PC2 ~ (Micro_PC1 + Micro_PC2 + Probiotic) × Environment

Model 2 (nutrient model):

SCFA + lactate_PC1 and SCFA + lactate_PC2 ~ (Nutrient_PC1 + Nutrient_PC2 + Probiotic) × Environment

where:

Probiotic—absence or presence of LGG supplementation;

Environment—DIVMS or volunteer.

Type III Analysis of Variance (ANOVA) was then applied to test main and interaction effects for the variables included in the models. An analysis of estimated marginal means was applied as a post hoc to Type III ANOVA. Given the single-donor, longitudinal design, this analysis was used as an exploratory means to identify factors associated with variation in PCA scores within available data. Results were not considered to provide population-level inference.

To assess the effect of outlier exclusion on the results, a sensitivity analysis was performed by repeating the main analysis on a complete dataset using conventional PCA and robust PCA (ROBPCA).

To assess the influence of the structure of the microbial community on various levels (α-diversity; the Bacillota-to-Bacteroidota ratio, formerly referred to as the Firmicutes-to-Bacteroidetes (F/B) ratio; most abundant microbial genera (MAG)) and nutrient concentration on SCFA levels in samples from each of the two studied environments (DIVMS and the volunteer), Spearman’s correlation analyses were run using LGG intervention-adjusted residuals. MAGs were defined as genera found at >1% abundance in at least 5 of 7 samples used in the statistical analysis.

All tests were performed at a significance level of 0.05. Multiple comparisons in Spearman’s correlations were corrected for by applying a Benjamini–Hochberg false discovery rate (FDR). Detailed outputs of statistical analyses and R scripts were deposited in an open data repository [46].

## 3. Results

### 3.1. Levels of Short-Chain Fatty Acids and Lactate In Vivo and In Vitro

**Figure 2:**
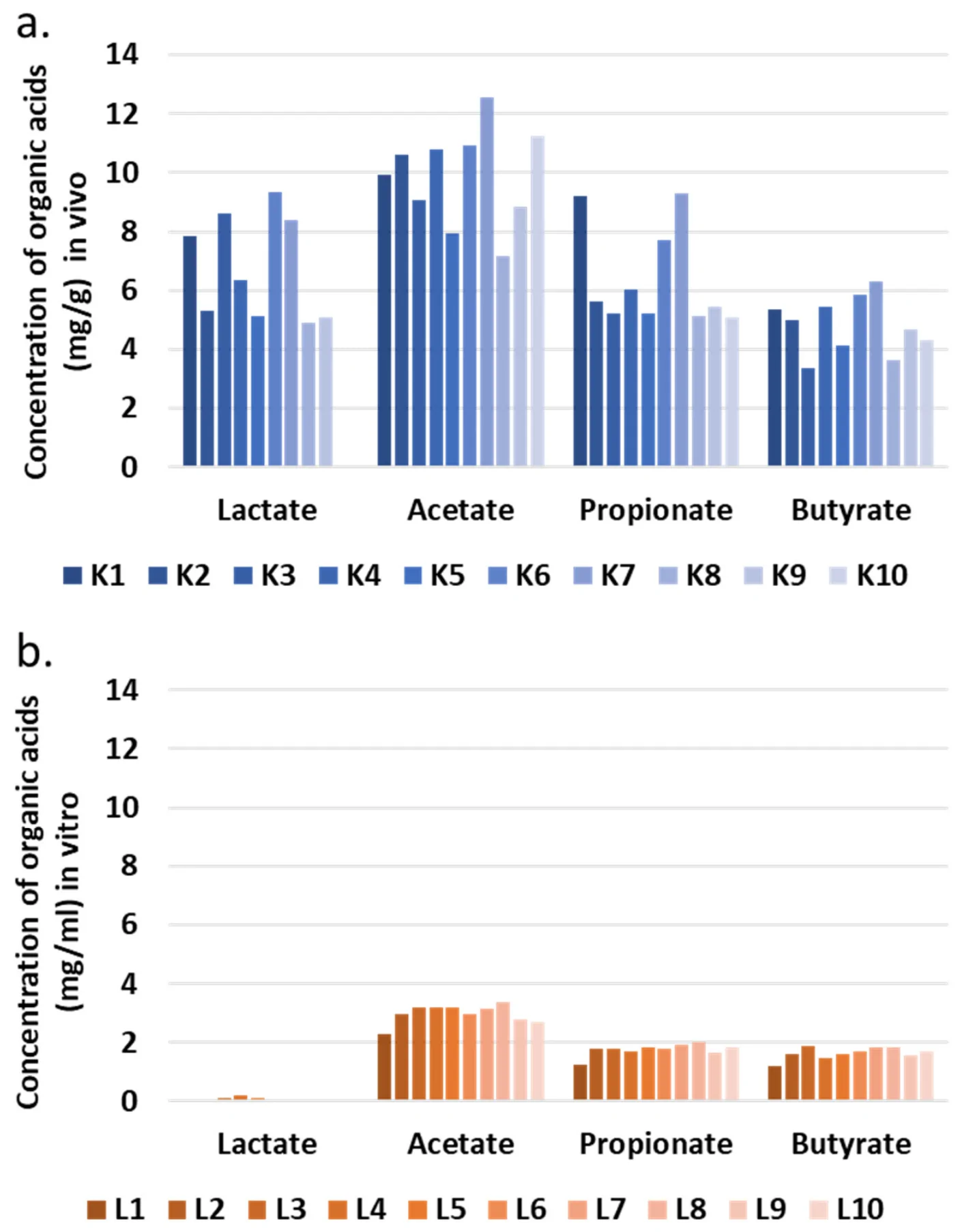
Concentration of lactate, acetate, propionate and butyrate in (**a**) volunteer fecal samples and (**b**) liquid DIVMS samples.

Figure 2 shows SCFA and lactate levels during the applied intervention.

In general, SCFA concentrations in liquid media for the DIVMS were several-fold lower than in the volunteer feces. The greatest difference was observed for lactate, which was detected in 9 out of 10 samples collected from the volunteer at levels ranging from 4.905 to 9.315 mg/g and in only four DIVMS samples at levels ranging from 0.067 to 0.215 mg/g. The greatest mean concentration over the experimental period in both in vivo and in vitro conditions was noted among SCFAs, for acetate, followed by propionate and butyrate.

No trends in changes in SCFA and lactate levels were observed over the experimental period. However, it was noted that in the DIVMS, lactate appeared only in four samples (L2–L5) taken during the probiotic intervention.

### 3.2. Influence of Interventions and Culturing Environment on Short-Chain Fatty Acids and Lactate Profiles

**Figure 3:**
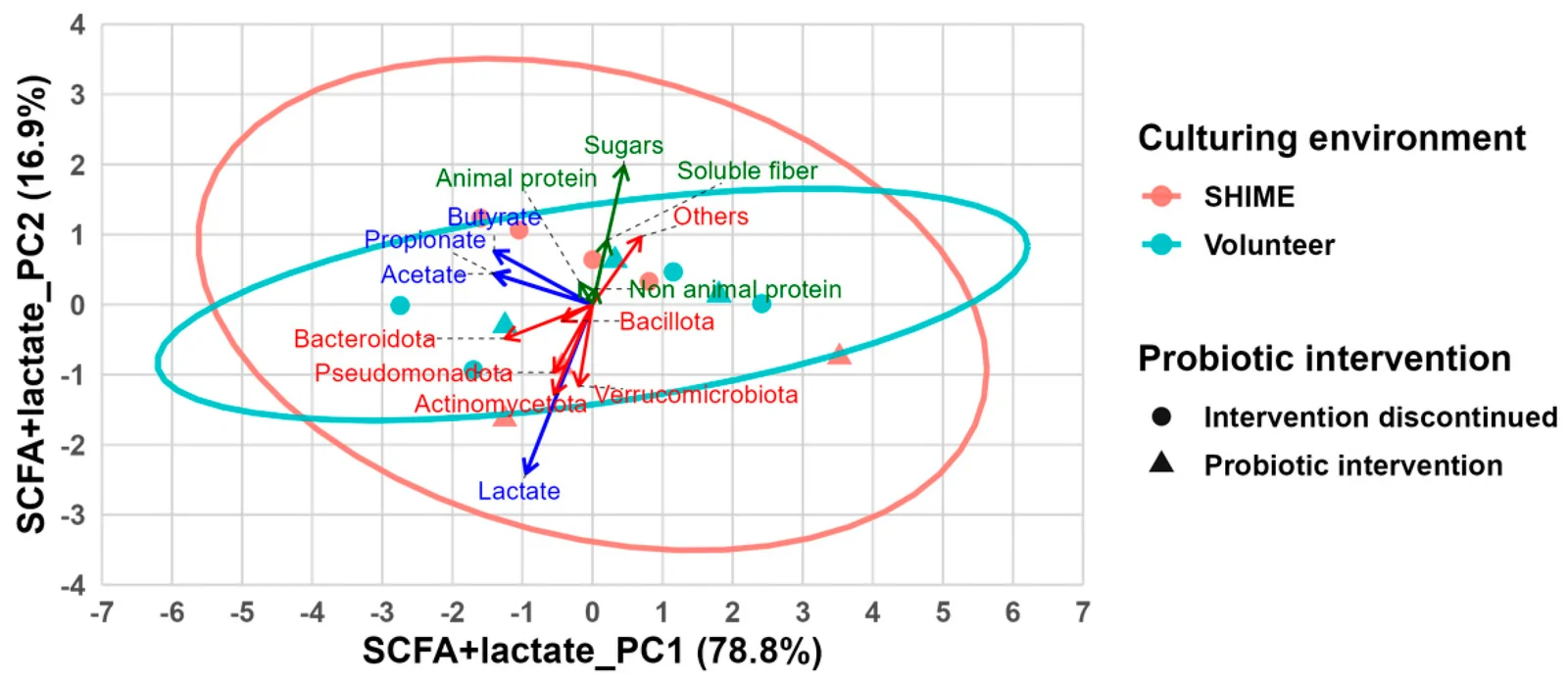
Biplot of principal components of the short-chain fatty acids and lactate (SCFA + lactate) in DIVMS and in the volunteer environments. Points represent samples, blue vectors show organic acid loadings, while red and green vectors express phyla and nutrient variables, respectively, fitted into SCFA + lactate PCA space based on their correlations with the principal component axes. Ellipses mark 95% confidence intervals around samples within each culturing environment.

The biplot shown in Figure 3 presents a summary of associations between organic acids, microbial phyla and nutrient variables in SCFA + lactate PCA space. Vectors representing nutrient concentrations 24 h before sampling and SCFA concentrations were oriented oppositely to the lactate vector. The SCFA vectors did not align well with any of the specific phyla abundance vectors. In contrast, the lactate concentration seemed to be associated particularly well with the abundance of Actinomycetota. In addition, vectors representing Pseudomonadota and Verrucomicrobiota abundance were closely aligned with the lactate concentration vector.

The type III ANOVA analyses revealed significant effects of the factors included in both models only along SCFA + lactate_PC2, but not along PC1. The largest proportion of model-explained variation was attributed to Probiotic (38% and 22% in the microbiota and nutrient models, respectively) and the interaction of Probiotic with Environment (31% and 24% variation explained, respectively). In addition, the Environment showed a significant independent effect on SCFA + lactate_PC2 variability in the nutrient model, explaining 16% of the model variation. Post hoc analysis of estimated marginal means revealed that the effect of Probiotic was significant only in the DIVMS environment, possibly due to previously noted lactate accumulation during the application of LGG intervention.

Sensitivity analysis demonstrated that the contribution of the Probiotic to SCFA + lactate_PC2 variability remained substantial in the full dataset (14–20% variance explained), although its statistical significance varied across analytical approaches. In contrast, the effect of Probiotic × Environment interaction was highly sensitive to outlier exclusion. In full dataset analyses, it was no longer statistically significant and accounted for only a few percent of variability.

**Figure 4:**
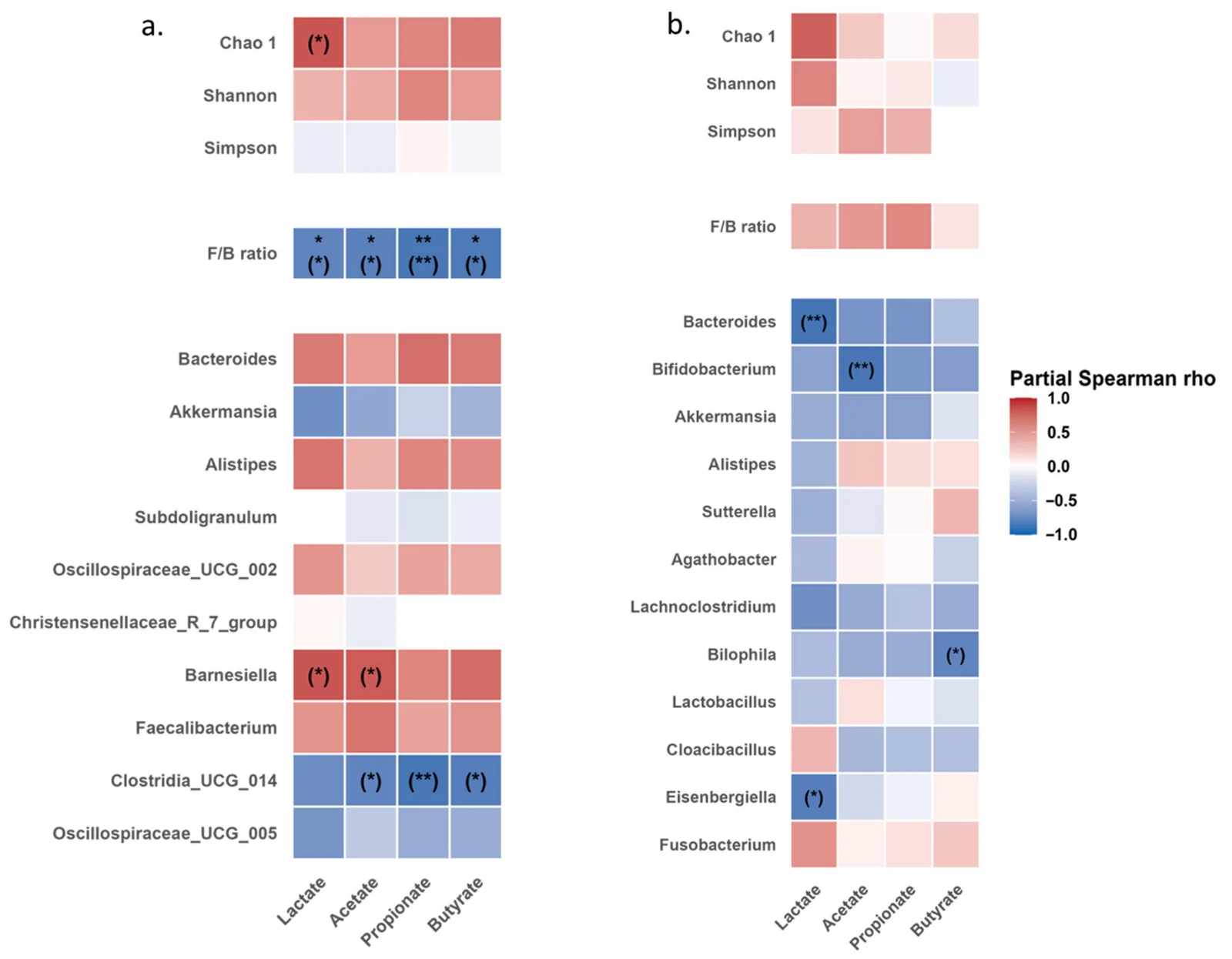
Heatmaps showing Spearman correlation coefficients between short-chain fatty acid (SCFA) + lactate concentrations and microbiota characteristics using *Lacticaseibacillus rhamnosus* GG (LGG) intervention-adjusted residuals in (**a**) fecal and (**b**) DIVMS samples. Microbiota characteristics include: (1) α-diversity indices, (2) the Firmicutes-to-Bacteroidetes (F/B) ratio, and (3) center log-ratio (CLR)-transformed relative abundances of the most abundant bacterial genera (defined as genera present at >1% relative abundance in at least 5 out of 7 analyzed samples within each environment). Statistical significance is indicated as * *p* < 0.05 and ** *p* < 0.01 in brackets for raw *p* values and without brackets for FDR-corrected values. FDR correction was applied separately for α-diversity indices and genus-level correlations; F/B ratio correlations were not corrected due to single-comparison testing.

To understand whether the microbiota characteristics at various levels of organization (α-diversity, F/B ratio and MAG), were associated with SCFA and lactate concentrations, Spearman correlation coefficients for probiotic-intervention-adjusted residuals were calculated and presented in Figure 4. Nominal positive correlations of Chao 1 index with lactate concentration and *Bernesiella* abundance with lactate and acetate concentrations were noted only in the samples taken from the human volunteer. All of these correlations became non-significant after FDR correction.

The only set of associations that remained significant under test conditions was negative correlations between the F/B ratio and all measured organic acids. Interestingly, these correlations were only found in the volunteer environment and not in the DIVMS. In the DIVMS these associations were not only not significant but also positive. An environment-driven difference in the SCFA–microbiota relationship was also noted for *Bacteroides*, whose abundance was positively associated with lactate and SCFA concentrations in the volunteer but showed a negative correlation with the concentrations of these organic acids in the DIVMS.

**Figure 5:**
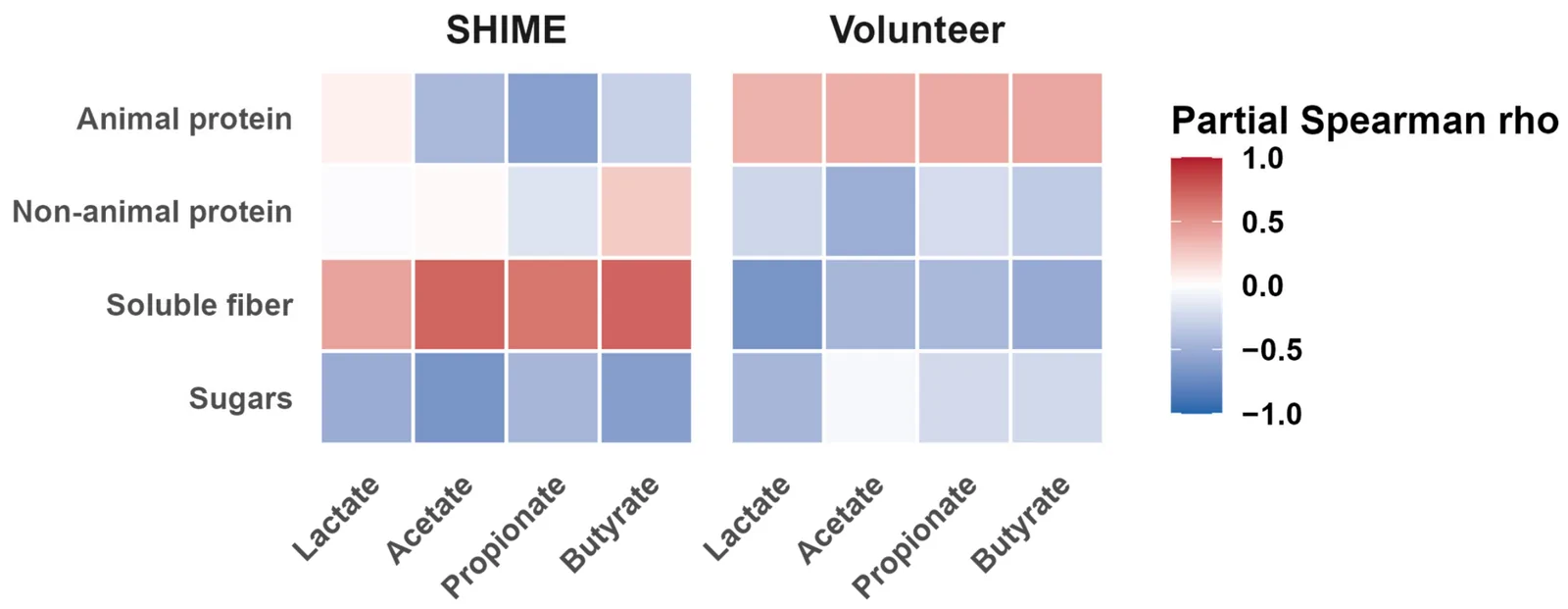
Heatmaps showing Spearman correlation coefficients between short-chain fatty acid (SCFA) + lactate concentrations and nutrient concentrations using *Lacticaseibacillus rhamnosus* GG (LGG) intervention-adjusted residuals in fecal and DIVMS samples. No statistical significance for the presented associations was determined.

Similarly, associations between individual nutrients and organic acid concentrations were studied by means of Spearman’s correlations (Figure 5). Results showed no statistically significant relationships between the variables. However, environment-specific patterns were observed. In the DIVMS, soluble fiber showed consistent positive correlations with all measured organic acids, whereas in the volunteer environment, a similar positive association pattern was observed mainly for animal protein.

## 4. Discussion

The relationship between fecal SCFAs and human microbiota is complex. On one hand, these compounds are produced by microbial fermentation, but on the other, they can also be consumed, transformed and metabolized by the microbiota. Human colonocytes are able to quickly and efficiently absorb most of the produced SCFAs [55]. Hence, high fecal SCFA levels were related to dysbiosis and some of the colonic dysfunctions, such as malabsorption or irritable bowel syndrome [10,56,57]. Some authors have shown that the α-diversity and fecal SCFA levels in humans were negatively correlated [10]. Here, correlations between α-diversity and SCFA concentrations were predominantly positive, but weak and not statistically significant. Thus, within this single-donor dataset, we have found no evidence of association between α-diversity and SCFA concentrations.

A positive correlation of fecal SCFAs with the F/B ratio was documented in a study with the participation of many human volunteers [58]. In the current study, we observed a converse directionality of this relationship in samples from our volunteer and no significant association in the DIVMS. SCFAs and lactate are produced by a range of different members of human microbiota that are assigned to both Bacillota and the Bacteroidota phyla [5]. Therefore, the F/B ratio alone may have a limited value in prediction of fecal SCFA levels. More studies with a repeated-measures design would certainly be useful to understand the reproducibility of the observation reported in this case study.

The microbiota changes compositionally when transitioning from human to in vitro conditions [34,40,59,60]. In samples used here, the relative abundance of identified taxa varied between the DIVMS and the volunteer on all levels from the phylum to the genus [40,45]. In particular, three genera that were at the highest abundances across samples supplied by the volunteer included *Bacteroides* (5–18%), *Subdoligranulum* (19–58%) and *Oscillospiraceae*_UCG_002 (3–11%), and in the DIVMS these were *Bacteroides* (40–52%), *Lachnoclostridium* (7–12%) and *Bifidobacterium* (4–10%). While *Bacteroides* are known producers of acetate and propionate, *Subdoligranulum* and *Oscillospiraceae* contribute butyrate, and *Bifidobacterium* and *Lachnoclostridium* acetate [61,62,63,64]. Nevertheless, no significant positive correlations between the abundance of these genera and SCFA levels in collected samples were noted. These observations suggest that the taxonomic profile of the microbiota alone may not be a reliable predictor of its metabolic behavior after transfer from in vivo to in vitro conditions. Studies suggest that the microbial metabolic activity may vary depending on substrate availability, physicochemical conditions and interactions with other community members, including cross-feeding networks [20,35,65].

Fecal SCFA and lactate concentrations reflect the balance between microbial production and consumption, together with host absorption and intestinal transit [4]. Despite clear, general differences in the makeup of the microbiota between the DIVMS and the volunteer, SCFA profiles remained similar between environments. Namely, acetate reached the highest concentrations among the three measured SCFAs in all samples, as expected based on human studies [36]. This observation may be explained by the previously documented functional redundancy, where distinct human microbiota communities were shown to sustain similar SCFA profiles [65].

On the other hand, lactate concentration was much higher in the volunteer’s compared to DIVMS samples. One possible explanation is the substantial increase in the mean dietary fiber intake during the intervention compared to habitual intake, from 14 to 38 g per day [34,45]. In contrast, the microbiota in the DIVMS received, on average, a very similar amount of fiber during the standard two-week stabilization phase preceding the experiment (7.7 g/L) and during the 28-day intervention (7.6 g/L) [34,43]. The abrupt increase in fiber intake in vivo may therefore have contributed to the higher concentration of fecal SCFAs and lactate observed in the volunteer compared to the DIVMS, despite contrary conclusions suggested in the subject literature [9]. While high fecal lactate concentrations, such as those observed in the volunteer’s samples in this study, are indicative of poor digestive health [6,30], here they were most likely a result of a dietary shift towards more balanced nutrition. It is known that a sudden increase in dietary fiber will lead to an increase in SCFA and lactate concentrations in excreted feces [11,66,67], even in such a short time as 24 h [8]. On the other hand, low lactate concentrations observed in the DIVMS are consistent with efficient lactate utilization observed in a healthy human microbiota [6,30]. In fact, in DIVMS media, during the experimental phase the total amount of fiber applied with the feed did not appreciably change compared to the stabilization phase, which could explain the lack of lactate accumulation, despite us manipulating the media composition between experimental days [17,34,40]. The approach used in this study to modify the DIVMS’s feed media to mimic the nutritional residues potentially reaching the colon of the volunteer is subject to uncertainty, because the amount of nutrients reaching the colon varies considerably among individuals [40,47] and here a mean estimate based on the available studies was applied. However, the accuracy of such predictions might be crucial and warrants further experimental investigation. Some authors point out that beyond the residual amount of fiber, its quality might be important for SCFA production. For example, Molly et al. found that among different fractions of fibers such as pectins, arabinogalactan and xylan, only starch levels influenced SCFA concentrations in the cultures of complex human microbiota [27].

Since nutritional residues, and especially dietary fiber, both soluble and insoluble fractions, seem to have a profound effect on lactate and SCFA concentrations in human feces [68], we expected to find some associations between nutrient fluctuation and organic acid levels. The results presented here (Figure 3) suggested an inverse association between nutrient availability, particularly sugars and lactate concentrations, with nutrient vectors oriented more closely towards the SCFA vectors. However, we failed to prove any statistically significant associations and only in the DIVMS, but not fecal samples, were positive correlations of soluble fiber concentrations with SCFA and lactate levels noted. In the volunteer, animal protein intake showed positive, although non-significant, correlations with organic acid concentrations. However, the literature indicates that dietary protein may have a negligible effect on fecal SCFA levels [69]. On the other hand, genera such as *Bacteroides* and *Clostridium* can utilize dietary protein and amino acids to synthesize branched short-chain fatty acids, particularly iso-butyric and iso-valeric acid [70], which were, however, beyond the focus of the current study.

Overall, our findings demonstrated that the culturing environment appeared to be more consistently associated with SCFA and lactate variability than either microbial composition or dietary variation, and that genus-level abundances correlated only weakly and inconsistently with metabolite pools across the in vitro and in vivo settings.

The probiotic supplementation was associated with altered SCFA and lactate profiles under in vitro conditions, while no corresponding effect was detected in vivo. Several mechanisms could contribute to this difference. In vivo, metabolic effects of the supplemented strain may be attenuated by host absorption, intestinal transit and interactions with established resident microbiota. In contrast, changes in microbiota composition that occur during adaptation in the in vitro environment may modify ecological niches and cross-feeding interactions, potentially increasing the detectability of responses to probiotics [12,71]. However, these mechanisms remain hypothetical, as LGG persistence, colonization and changes in cross-feeding interactions were not directly investigated in the present study.

While discussing the findings, it needs to be noted that although the study was designed with care, it is not without limitations. The repeated-measures design was intended to capture temporal dynamics of organic acid fluctuations and compare these dynamics between in vitro and in vivo conditions. This approach enabled assessment of changes over time in each of the tested conditions. However, the lack of biological replication restricts the generalizability of the findings. Therefore, the results of this study support the proposed hypothesis within the applied experimental setting. To our knowledge, this is the first study monitoring organic acid levels in the feces of the donor and distal colonic liquid from the dynamic simulator during a parallel intervention. The relevance of the present case study therefore lies not in demonstrating a population-level effect of transfer to the in vitro conditions, but in showing that donor-specific functional responses cannot be assumed to remain preserved after such transfer. Further studies encompassing multiple individual donors and parallel in vitro simulations would be desirable to confirm these findings and establish their broader applicability.

## 5. Conclusions

In this study, we found that despite substantial compositional differences between the donor and DIVMS microbiota, the broad SCFA profile was preserved across environments, with acetate remaining the predominant SCFA. While this typical behavior of the human microbiota, known from multiple human and DIVMS studies, was replicated here, the absolute metabolite concentrations, particularly lactate, and microbiota–metabolite associations were strongly environment-dependent. In addition, LGG supplementation was associated with changes in SCFA profiles in the DIVMS, whereas no corresponding effect was detected in the volunteer. These findings are important in the context of further understanding of the limitations of the current state-of-the-art microbiota simulation systems. While the DIVMS may allow for preservation of some broad community-level metabolic characteristics, such as the SCFA proportions, the donor-specific functional response cannot be assumed. The functional characteristics of an individual microbiota with which the system is inoculated might be altered during adaptation. On the other hand, preservation of taxonomic composition does not necessarily imply functional transferability, as the metabolic contribution of individual taxa may change in response to substrate availability, community interactions and the physicochemical conditions of the in vitro environment. Hence, further studies on modifications to standard protocols aiming at preservation of the traits of particular microbiota would be required. Such studies should adopt multifactorial and multi-omics approaches that integrate sequencing, metabolomics and data from controlled interventions [72]. Datasets can then be incorporated into community-scale metabolic models and machine-learning frameworks to predict individual SCFA responses [73,74]. The predictive power of these approaches could be further improved by combining them with personalized, donor-specific in vitro reactors. Such systems should be explicitly calibrated to account for the structural and functional changes that arise during the transition from in vivo to in vitro conditions. This integrated strategy may enable the prediction of host-specific metabolic outcomes and, ultimately, support the rational design of personalized dietary and probiotic interventions [73,74].

In vitro studies on individual microbiotas are important in the context of the development of personalized nutritional and medicinal strategies, whose effectiveness is heavily dependent on the microbiota. Standard stabilization protocols in the DIVMS may selectively favor microbial communities adapted to reactor conditions, potentially reducing some donor-specific compositional and functional characteristics. This may mean that effects measured in microbiota seen from applied interventions are not accurately estimated on the individual level, which seems to be supported by the results relating to the organic acid profile and LGG supplementation here. Overall, the translational value of observations contained in this study should be considered in the context of a persistent gap in personalized microbiome science. More research reporting changes in microbiota transition after inoculation into dynamic in vitro systems and on the development of a stabilization protocol would be crucial to fill this gap.

## Data Availability

The dataset that allowed us to generate results reported in this study is contained in an open repository under https://doi.org/10.5281/zenodo.14974398 (accessed on 1 August 2026), https://doi.org/10.5281/zenodo.16751627 (accessed on 1 August 2026; also for two previous reports) and https://zenodo.org/records/22086679 (accessed on 24 August 2026 exclusively the current manuscript).

## Abbreviations

The following abbreviations are used in this manuscript:

ANOVA	Analysis of Variance
CFU	Colony-forming units
CLR	Centered log-ratio transformation of compositional data
DIVMS	Dynamic in vitro microbiota cultivation simulator
DNA	Deoxyribonucleic acid
FDR	False Discovery Rate
F/B ratio	Firmicutes-to-Bacteroidetes ratio
HPLC	High-performance liquid chromatography
LGG	*Lacticaseibacillus Rhamnosus* Strain GG
L-SHIME	Luminal Simulator of Human Gastrointestinal Microbial Ecosystem
MAG	Most Abundant Genera
PC	Principal Component
PCA	Principal Component Analysis
PES	Polyethersulfone
rRNA	Ribosomal ribonucleic acid
SHIME	Simulator of Human Gastrointestinal Microbial Ecosystem
UV/Vis	Ultraviolet–visible
